# P-148. A Practical Antibiogram for Community-Acquired Gastroenteritis in British Columbia, Canada

**DOI:** 10.1093/ofid/ofaf695.374

**Published:** 2026-01-11

**Authors:** Eugene Yeung

**Affiliations:** University of British Columbia

## Abstract

**Background:**

Hospital microbiology laboratories do not routinely allow testing for community gastrointestinal stool pathogens in hospitalized patients who develop diarrhea after day 3 of hospitalization. In contrast, community microbiology laboratories routinely test and collect data of gastrointestinal stool pathogens, including their antimicrobial susceptibility profiles. The current study aimed to generate a practical antibiogram based on bacterial stool culture and susceptibility results collected in regional community microbiology laboratories in British Columbia (BC), Canada.

**Methods:**

LifeLabs BC microbiology laboratories, connected with 129 collection centres in urban and rural communities in the province, provided the laboratory data for the fecal bacterial pathogens. An audit was conducted from January 1, 2020 to December 31, 2023. Calculations were performed as per the Clinical and Laboratory Standards Institute M39 document (2022).

**Results:**

**Table:** 

ORGANISM	Ampicillin	Trimethoprim-Sulfamethoxazole	Ciprofloxacin	Tetracycline	Azithromycin	Ceftriaxone
Aeromonas species, % susceptible	R	96	99	99		98
n =		677	664	664		774
Campylobacter jejuni/coli, % susceptible	R	R	55	65	100	
n =			55	55	59	
Salmonella species#, % susceptible	91	94	85		@	99
n =	326	292	318			338
Shigella species, % susceptible	19	12	21		31	91
n =	243	225	230		113	243
Vibrio species, % susceptible	R	97	100	97	@	
n =		32	37	37		
Yersinia species, % susceptible	R	100	99	99		100
n =		822	878	927		980

R = The organism is intrinsically resistant to the antibiotic indicated OR is not recommended due to poor clinical response and/or poor activity

@ = Azithromycin may be a reasonable treatment option for *Salmonella* and *Vibrio* infections when clinically indicated; however, LifeLabs has not tested enough isolates to generate a meaningful antibiogram.

**Conclusion:**

Multidrug and extended-drug resistant *Salmonella* species do not appear to be a current problem in British Columbia. Choosing the optimal oral antimicrobial agent for *Shigella* species is a challenge as sensitivities of oral agents were < 50%.

**Disclosures:**

All Authors: No reported disclosures

